# Approaches to identify scenarios for data science implementations within healthcare settings: recommendations based on experiences at multiple academic institutions

**DOI:** 10.3389/fdgth.2025.1511943

**Published:** 2025-03-14

**Authors:** Lillian Sung, Michael Brudno, Michael C. W. Caesar, Amol A. Verma, Brad Buchsbaum, Ravi Retnakaran, Vasily Giannakeas, Azadeh Kushki, Gary D. Bader, Helen Lasthiotakis, Muhammad Mamdani, Lisa Strug

**Affiliations:** ^1^ Department of Paediatrics, The Hospital for Sick Children, Institute of Health Policy Management & Evaluation, University of Toronto, Toronto, ON, Canada; ^2^ Department of Computer Science, Vector Institute for Artificial Intelligence, University Health Network, University of Toronto, Toronto, ON, Canada; ^3^ Institute of Health Policy Management & Evaluation, University Health Network, University of Toronto, Toronto, ON, Canada; ^4^ Department of Medicine, Department of Laboratory Medicine and Pathobiology, and Institution of Health Policy Management & Evaluation; St. Michael’s Hospital, Unity Health Toronto, University of Toronto, Toronto, ON, Canada; ^5^ Department of Psychology, Rotman Research Institute, Baycrest Centre, University of Toronto, Toronto, ON, Canada; ^6^ Division of Endocrinology, Lunenfeld-Tanenbaum Research Institute, Mount Sinai Hospital, Toronto, ON, Canada; ^7^ Women’s College Research Institute, Women’s College Hospital, Toronto, ON, Canada; ^8^ Institute of Biomedical Engineering, Holland Bloorview Kids Rehabilitation Hospital, University of Toronto, Toronto, ON, Canada; ^9^ Department of Molecular Genetics, Temerty Faculty of Medicine, Toronto, ON, Canada; ^10^ Data Sciences Institute, University of Toronto, Toronto, ON, Canada; ^11^ Temerty Faculty of Medicine, Centre for Artificial Intelligence Education and Research in Medicine, Unity Health Toronto, University of Toronto, Toronto, ON, Canada

**Keywords:** machine learning, data sciences, healthcare, use case, scenario

## Abstract

**Objectives:**

To describe successful and unsuccessful approaches to identify scenarios for data science implementations within healthcare settings and to provide recommendations for future scenario identification procedures.

**Materials and methods:**

Representatives from seven Toronto academic healthcare institutions participated in a one-day workshop. Each institution was asked to provide an introduction to their clinical data science program and to provide an example of a successful and unsuccessful approach to scenario identification at their institution. Using content analysis, common observations were summarized.

**Results:**

Observations were coalesced to idea generation and value proposition, prioritization, approval and champions. Successful experiences included promoting a portfolio of ideas, articulating value proposition, ensuring alignment with organization priorities, ensuring approvers can adjudicate feasibility and identifying champions willing to take ownership over the projects.

**Conclusion:**

Based on academic healthcare data science program experiences, we provided recommendations for approaches to identify scenarios for data science implementations within healthcare settings.

## Background

Healthcare institutions rely upon data to influence decision making at the organizational level and to improve patient outcomes (1). Examples range from simple reports to rule- or machine learning- (ML) based algorithms. Constraints to using data to drive decision making include, but are not limited to, challenges with data access, security and privacy restrictions, need to ensure patient safety and address ethical aspects, fragmented data ecosystems and funding/resource limitations. These remain open challenges in implementing data science approaches. It is important to identify solutions to these challenges to accelerate data science-directed advancements in healthcare.

Scenario identification (or “use case” identification) is a specific challenge for data sciences in the clinical context because of the need to curate solutions and optimize use of scarce resources. Scenario refers to both the operational or clinical problem in addition to the specific setting in which the data will be applied. For example, sepsis is an example of a clinical problem whereas sepsis prediction during admission to a general ward is an example of a scenario.

There are several papers that have described frameworks for healthcare institutions to develop and implement data science solutions, including ML (2–8). However, most propose solutions that are generated from a theoretical or a single institution perspective. In contrast, we hypothesized that building upon the experiences of several academic health sciences institutions implementing data science solutions would reveal more general patterns associated with successful implementations. We define success as scenarios leading to institutional adoption, favorable clinical or operational impact and continued implementation. Successful scenarios include early warning systems for clinical deterioration (9, 10), mortality (11) prediction, and sepsis (12) prediction as examples. Our objective was to describe successful and unsuccessful approaches to identify scenarios for data science implementations within healthcare settings and to provide recommendations for future scenario identification procedures.

## Materials and methods

The Data Sciences Institute (DSI) is a multi-divisional, tri-campus and multidisciplinary hub for data science activity at the University of Toronto. The DSI mission is to accelerate the impact of data sciences across disciplines to address pressing societal questions and drive positive social change. They define data sciences as the science of collecting, manipulating, storing, visualizing, learning from and extracting useful information from data in a reproducible, fair and ethical way. DSI partners with seven healthcare institutions in Toronto and collaborates with the Temerty Faculty of Medicine Centre for Artificial Intelligence Education and Research in Medicine (T-CAIREM). T-CAIREM supports a community of over 1,500 members focused on research, education and infrastructure for artificial intelligence in health care.

The DSI and T-CAIREM co-hosted a one-day in-person workshop with representation from the following seven DSI-affiliated healthcare institutions: Baycrest Centre, Holland Bloorview Kids Rehabilitation Hospital, Mount Sinai Hospital, The Hospital for Sick Children, Unity Health Toronto, University Health Network and Women's College Hospital. The workshop was held at the DSI offices on April 5, 2024. Each institution was asked to provide a brief introduction to their data science program and to provide an example of a successful and unsuccessful approach to scenario identification at their institution. Each presentation was followed by a question and answer period.

Once all presentations were completed, attendees first outlined some general observations about the potential utility and limitations of experience reflections from this group. Next, we outlined general considerations required to develop and implement data science solutions in healthcare, where the focus of the present meeting was to discuss scenario identification approaches. Finally, the discussion centered on common observations across experiences with successful and unsuccessful approaches to identify scenarios for data science implementations.

Notes were taken during the meeting by one author (HL). Using the presentations themselves and the meeting notes, content analysis (13) was performed by a single reviewer (LS). These results were evaluated and revised by a second reviewer (HL). If discrepancies were identified, the two reviewers met to come to consensus. Summarized results were then reviewed and confirmed by all authors.

## Results

We observed that sharing of experiences was useful for learning and collaborating, with the potential to accelerate each hospital's program by leveraging insights made at other institutions. We also observed that a synthesis of approaches that are consistently successful and those that are consistently unsuccessful is likely to be generalizable across many settings. High-level approaches are more likely to be generalizable rather than the specific plans to implement, which will typically be driven by local circumstances.

Table 1 describes the general considerations required to develop and implement data science solutions in healthcare, where scenario identification was the focus of the workshop. Table 2 describes common observations with successful and unsuccessful approaches to identify scenarios. Examples were shown by idea generation and value proposition, prioritization, approval and champions. Table 2 also provides recommendations related to each of these areas.

**Table 1:** General considerations required to implement data science solutions in healthcare.

Considerations to implement data science solutions
Scenario identification: idea generation and value proposition, prioritization and approval^a^
Data acquisition: access and governance
Data infrastructure including ongoing support and quality assurance
Solution design and/or procurement, including model development
Pre- and post- deployment planning and monitoring including education, change management including implementation science considerations and maintenance
Evaluation of impact on patient or operational outcomes and return on investment

^a^ Focus for this manuscript.

**Table 2:** Common observations with successful and unsuccessful approaches to identify scenarios for data science implementations.

Theme	Success examples	Unsuccessful examples	Recommendations
Idea generation and value proposition	Promote a portfolio of ideas, allowing many to propose different scenarios and solutions Clear value proposition Using data-driven approaches to problem identification	Multiple groups proposing same scenario and solution working in silos Operational mindset that does not permit innovation or change	Require approval from target users who can identify and harmonize duplicate efforts Endorsement by both clinical and operational leadership Allow some risk tolerance
Prioritization	Aligned with organizational priorities Successfully solves a real operational or clinical problem	Focus on research without articulation of clinical or operational benefit	Articulate strategic priority Align research energy and culture with problems prioritized by clinicians and leaders
Approval	Single or small group of individuals with knowledge and commitment to evaluate proposal	Large executive committee that may lack knowledge to identify promising and feasible solutions or may not invest energy to understand proposal	Small group Can judge problem importance, feasibility, return on investment Need prioritization framework
Champions	Individuals at senior level at decision-making table as well as implementation champions Willing to take ownership Knowledgeable	No one willing to “own” solution or be accountable for poor outcomes	Individual or group who takes responsibility for project success or failure Requires representation from target users

### Idea generation and value proposition

In the context of scenario identification, we recommend that there is a mechanism, within an organization, that allows many ideas to be proposed for downstream prioritization. Ideas can be generated and shaped from a wide variety of sources spanning operational and clinical needs to an individual clinician's or researcher's ideas. To address unmet needs, an operational perspective that prioritizes aligned innovation or improvement is required. We also recognized the importance of balancing risk, and that some risk tolerance should be promoted as long as it is monitored and it is balanced against potential benefit. A critical component of scenario identification is clear articulation of the value proposition and anticipated return on investment for scenario development and deployment. In identifying scenarios, it is important to use local data to ensure problems or outcomes are sufficiently common to justify institutional investment.

An unsuccessful example was when the same scenario was being proposed by multiple groups for the same target user, without adequate engagement of the actual target user. These different groups might represent different operational, clinical or research perspectives. Identifying duplicative siloed efforts is important; target user may be in the best position to recognize and reconcile them.

### Prioritization

It is important to distinguish between approval to explore in the research phase vs. approval to implement. We suggest that if there are no or limited resource implications, researchers should be encouraged to innovate and identify solutions as freely as possible, while adhering to regulatory and privacy frameworks. Prioritization should occur prior to the point of clinical implementation, or when there are implications for institutional resources or impact. Ultimately, the prioritization of initiatives will be driven by a balance between the anticipated benefits and the resources required to develop, deploy and maintain the solution.

Prioritization is a critical process that should consider limited resources, organizational strategy, prioritized improvement efforts and overall cost-benefit. While it is important that prioritization aligns with operational and clinical needs, how to actually prioritize specific projects and who performs the prioritization will likely differ between institutions. Institutions should promote alignment of research energy and culture with problems prioritized by clinicians and leaders where feasible. The specific roles of relevant clinical and operational leaders will vary across institutions.

### Approval

The mechanism to approve scenarios was particularly heterogeneous. The group agreed that the approval process must take into account what operational or clinical scenarios are important to the organization as well as determine the feasibility of a data science approach. The approval process must also be able to assess likely return on investment. Successful examples included single or a small number of knowledgeable individuals to approve scenario identification. An unsuccessful example was when large executive groups made the decision of which data science solutions were to proceed as they may not have the requisite knowledge to be able to judge clinical impact or data science feasibility. With large groups, there may be few members who engage in the discussion or make the effort to fully understand the proposal, its feasibility and likelihood for success.

### Champions

It is important that there is an individual or group of individuals who are willing to take ownership over the project implementation and to be accountable for the measured success and failure. This ownership must occur throughout the various stages of the project. The specific professional characteristics of champions will vary depending on the specific scenario and across institutions. These individuals are likely to be at both the clinical and executive levels. In contrast, projects were not successful when no one was willing to take responsibility for them.

## Discussion

We summarize successful and unsuccessful approaches to identify scenarios for data science implementations in healthcare. Observations were coalesced to idea generation and value proposition, prioritization, approval and champions. Successful experiences included promoting a portfolio of ideas, articulating value proposition, ensuring alignment with organization priorities, ensuring approvers can adjudicate feasibility and identifying champions willing to take ownership over the projects.

While these observations arose out of real-world experiences, we recognize that there are many appropriate ways to generate and approve scenarios and our observations are unlikely to be applicable to all settings. Nonetheless, despite different institutions and patient populations (e.g., spanning neonatal to geriatric), observed commonalities support some generalizability. We hope that sharing our successes and challenges can contribute to the broader discourse around data-driven decision making in healthcare.

Scenario identification, prioritization and approval is only one step in data science implementations in healthcare. Future workshops could focus on identifying shared experiences related to other aspects such as data access and governance as an example. Another important aspect that warrants further exploration is approaches that promote successful implementations and sustainability post deployment.

The strength of this report lies in the number and heterogeneity of contributing organizations. However, this report is limited as all institutions belonged to the same umbrella networks, namely DSI and T-CAIREM. Factors that influence decisions at academic centers in Toronto may be different than in other places. In particular, these findings may not be generalizable to very different contexts or countries. Further, despite the number and heterogeneity of organization involved, they do not cover all possible types of healthcare contexts and thus continued analysis of diverse settings will be beneficial.

In summary, based on academic healthcare data science program experiences, we provided recommendations for approaches to identify scenarios for data science implementations within healthcare settings. Future efforts should focus on other requirements to successfully deploy data sciences solutions in healthcare.

## Data Availability

The original contributions presented in the study are included in the article/supplementary material, further inquiries can be directed to the corresponding author.
